# Vitamin D Signaling in the Context of Innate Immunity: Focus on Human Monocytes

**DOI:** 10.3389/fimmu.2019.02211

**Published:** 2019-09-13

**Authors:** Carsten Carlberg

**Affiliations:** School of Medicine, Institute of Biomedicine, University of Eastern Finland, Kuopio, Finland

**Keywords:** vitamin D, VDR, epigenome, transcriptome, gene regulation, vitamin D target genes, monocytes, PBMCs

## Abstract

The vitamin D_3_ metabolite 1α,25-dihydroxyvitamin D_3_ (1,25(OH)_2_D_3_) activates at sub-nanomolar concentrations the transcription factor vitamin D receptor (VDR). VDR is primarily involved in the control of cellular metabolism but in addition modulates processes important for immunity, such as anti-microbial defense and the induction of T cell tolerance. Monocytes and their differentiated phenotypes, macrophages and dendritic cells, are key cell types of the innate immune system, in which vitamin D signaling was most comprehensively investigated *via* the use of next generation sequencing technologies. These investigations provided genome-wide maps illustrating significant effects of 1,25(OH)_2_D_3_ on the binding of VDR, the pioneer transcription factors purine-rich box 1 (PU.1) and CCAAT/enhancer binding protein α (CEBPA) and the chromatin modifier CCCTC-binding factor (CTCF) as well as on chromatin accessibility and histone markers of promoter and enhancer regions, H3K4me3 and H3K27ac. Thus, the epigenome of human monocytes is at multiple levels sensitive to vitamin D. These data served as the basis for the chromatin model of vitamin D signaling, which mechanistically explains the activation of a few hundred primary vitamin D target genes. Comparable epigenome- and transcriptome-wide effects of vitamin D were also described in peripheral blood mononuclear cells isolated from individuals before and after supplementation with a vitamin D_3_ bolus. This review will conclude with the hypothesis that vitamin D modulates the epigenome of immune cells during perturbations by antigens and other immunological challenges suggesting that an optimal vitamin D status may be essential for an effective epigenetic learning process, in particular of the innate immune system.

## Introduction

**Figure 1:**
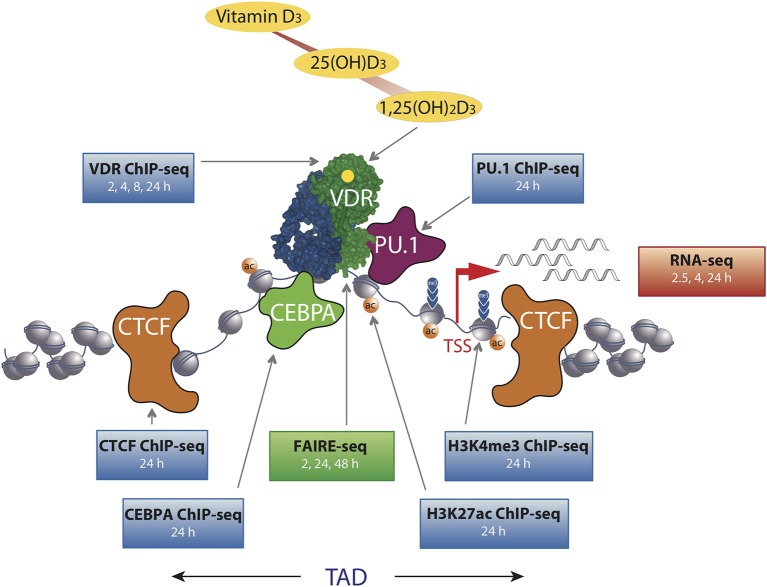
The chromatin model of vitamin D signaling. The model was defined by epigenome- and transcriptome-wide data obtained in THP-1 cells. CTCF proteins define left and right TAD borders, in which vitamin D target genes (red arrow, measured by RNA-seq) are activated by VDR [activated by 1,25(OH)_2_D_3_] binding to enhancer regions. The pioneer transcription factors CEBPα and PU.1 help VDR in binding to accessible genomic DNA (measured by FAIRE-seq). This paralleled with changes in markers of active TSS regions (H3K4me3) and active chromatin (H3K27ac). The genome-wide binding of VDR, CTCF, PU.1, CEBPα, and histone markers were determined by ChIP-seq in three biological repeats. The time of 1,25(OH)_2_D_3_ stimulation is indicated for each dataset.

Vitamin D_3_ is an evolutionary very old molecule that is produced from the direct cholesterol precursor 7-dehydrocholesterol in a non-enzymatic reaction using energy provided by the UV-B component of sunlight (1). Thus, every species that exerts cholesterol biosynthesis and is exposed to sunlight should be able to synthesize vitamin D_3_. The molecule itself is biologically inert, but when it is converted to 25-hydroxyvitamin D_3_ (25(OH)D_3_) and then to 1α,25-dihydroxyvitamin D_3_ (1,25(OH)_2_D_3_), it acts as a nuclear hormone. The jawless fish lamprey is the oldest known species that some 550 million years ago evolved with the transcription factor VDR a nuclear receptor

that gets activated by 1,25(OH)_2_D_3_ at sub-nanomolar concentrations (2). After the manifestation of VDR, vitamin D turned from a product of UV-B absorption, i.e., the output of a radiation protection pathway as found in plankton, to an endocrine molecule in higher species (3). Thus, vitamin D has via its metabolite 1,25(OH)_2_D_3_ direct effects on gene regulation (Figure 1). In human, the main sites of 1,25(OH)_2_D_3_ production for endocrine purposes are proximal tubule cells of the kidneys, but for para- and autocrine use also monocytes, macrophages, and dendritic cells of the innate immune system and other cell types in skin and bone are able to produce the nuclear hormone (4).

Since vitamin D_3_ can be synthesized endogenously in human skin (5), the term “vitamin” seems not to be appropriate. However, compared to the past, humans spend far more time indoors, largely cover their skin by textile when being outdoors and often live at latitudes where during winter UV-B radiation is too low for many months, there is insufficient endogenous vitamin D_3_ production, i.e., under these conditions vitamin D_3_ is an essential micronutrient (6). Average human diet is low in vitamin D, so that dietary products, such as milk, margarine and juices, are fortified and direct vitamin D supplementation via pills is recommended in winter months (7). Interestingly, already more than 100 years ago cod-liver oil as well as UV-B exposure had been proposed for the protection against rickets (an infant bone malformation disease) as well as for the treatment of tuberculosis (an infectious disease caused by intra-cellular bacteria) (8, 9). Thus, vitamin D deficiency causes not only bone disorders (10) but also affects the protective roles of the molecule against a large number of other diseases (11). The autoimmune disease multiple sclerosis is the most prominent example, which may be largely preventable by a sufficient vitamin D status (12). This status is defined via the serum concentrations of the most stable vitamin D metabolite, 25(OH)D_3_, which for good bone health should be 50 nM (13), but also levels of 75 nM or more are suggested (14). Accordingly, instructions for daily supplementation with vitamin D_3_ range from 10 to 50 μg (400–2,000 IU). However, these population-wide recommendations do not take inter-individual variations into account, such as a different molecular response to vitamin D, which are expressed by the vitamin D response index (15). As discussed below in more detail, this index can be determined *via* the genome-wide response of peripheral blood mononuclear cells (PBMCs) to an *in vivo* challenge with vitamin D_3_ (16).

In extension to a recent summary on the nutrigenomic role of vitamin D (17), the aim of this short review is to present the epigenome-wide impact of the nuclear hormone in relation to immunity. Special attention is given to human monocytes and PBMCs serving as *in vitro* and *in vivo* model systems for vitamin D signaling.

## Vitamin D and the Epigenome

**Figure 2:**
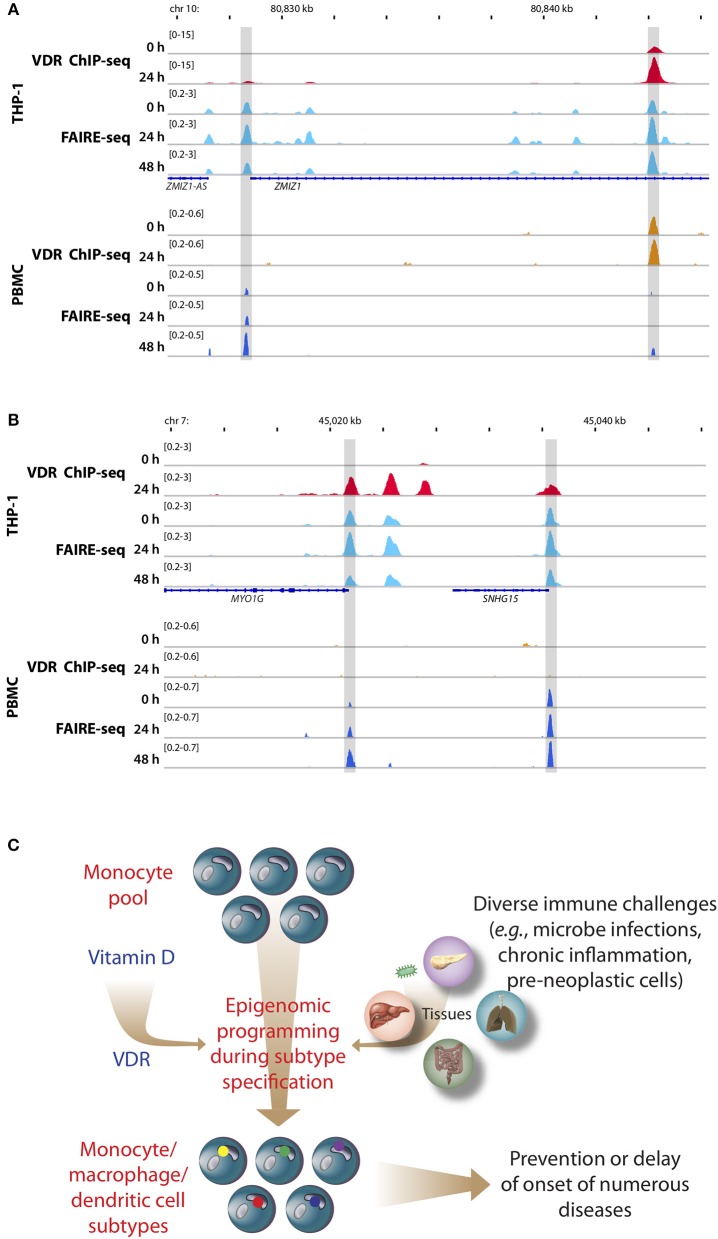
Memory hypothesis. VDR binding and chromatin opening of the loci of the genes *ZMIZ1*, **(A)** and *MYO1G* **(B)** *in vitro* (THP-1) as well as *in vivo* (PBMCs). THP-1 cells were stimulated for 0, 24, and 48 h with 1,25(OH)_2_D_3_ and VDR ChIP-seq and FAIRE-seq were performed (22). In a comparable *in vivo* experiment an individual was challenged with a vitamin D_3_ bolus (2,000 μg) and PBMCs were isolated before (day 0) and at days 1 (24 h) and 2 (48 h) (23). The peak tracks represent merger of each three biological repeats. Gene structures are shown in blue. Different types of immune challenges program the epigenome of the pool of human monocytes, which “memorize” these encounters in form of differently programmed epigenomes leading to subtype differentiation (bottom, differently colored dots of in nuclei of monocyte subpopulations, **C**). The recently discovered epigenome modulating effect of vitamin D [via the VDR (24)] modulates on multiple levels this epigenetic programming process. The stabilization of the epigenomes of the subtypes of monocytes, macrophages and dendritic cells by vitamin D can prevent or delay the onset of common age-related diseases.

Chromatin is a complex of histone proteins and genomic DNA (18, 19) that by default largely restricts the access of RNA polymerases to promoter regions and of transcription factors to enhancer regions. Therefore, in a differentiated cell only some 200,000 genomic loci are accessible (20). The epigenome comprises genome-wide information represented by covalent and structural modifications of chromatin, such as cytosine methylation, post-translational modifications of histone proteins and 3D structure of the nucleus, that do not involve any alterations in the sequence of genomic DNA (21). Epigenetic programming is a memory creating event that during embryogenesis and cellular differentiation, such as of monocytes after immune challenges (Figure 2C), determines the specialized roles of terminally differentiated cells *via* changes of their epigenome (25). In these cases epigenetic programming is irreversible and leads to static outcomes, in order to keep the identity of tissues and cell types. Thus, the epigenome largely determines gene expression and the functional profile of a cell; i.e., alternations of the epigenome precede those of the transcriptome.

A number of diet-derived metabolites, such as resveratrol, genistein, curcumin and polyphenols from fruits, vegetables, spices, teas and medicinal herbs, can affect the activity of chromatin modifiers and transcription factors (17, 26). Chromatin modifiers are nuclear enzymes that catalyze epigenetic modifications, such as DNA methylation as well as histone acetylation and methylation, while chromatin remodelers are another class of nuclear enzymes that change the position and composition of nucleosomes. VDR communicates in a ligand-dependent fashion both with chromatin modifiers, such as lysine demethylase 6B (KDM6B) (27), as well as with chromatin remodelers, such as bromodomain containing 7 (BRD7) (28). This explains how vitamin D can significantly change the intensity of histone markers for active chromatin, H3K27ac, as well as those for active transcription start sites (TSSs), H3K4me3, as observed by chromatin immunoprecipitation sequencing (ChIP-seq) in THP-1 human monocytic leukemia cells (29, 30) (Figure 1). The epigenome of these cells responds to a stimulation with 1,25(OH)_2_D_3_ at the loci of more than 500 promoters and 2,500 enhancers. Moreover, the method of formaldehyde-assisted isolation of regulatory elements sequencing (FAIRE-seq) monitored in the same cellular system that vitamin D changes the accessibility of some 4,500 chromatin loci (out of some 100,000 in total) at a given time point (22). Thus, a stimulation with vitamin D represents a cellular perturbation that results in changes of the epigenome and in this way affects the epigenetic memory of the cell (24). However, in contrast to epigenetic programming during cellular differentiation, many of these epigenetic memorizing events are dynamic; i.e., they persist only for a shorter time period and are reversible.

In human cells, the cistrome of VDR, i.e., the genome-wide binding pattern of the transcription factor, was determined by the ChIP-seq in lymphocytes (31), colorectal cancer cells (32), hepatic stellate cells (33), prostate cells (34), macrophage-like cells (35) and most comprehensively in monocytes (36, 37). In all these *in vitro* cell culture models stimulation with ligand resulted in a 2- to 10-fold increase in VDR binding sites; i.e., the significantly enhanced VDR cistrome represents the most eminent response of the human epigenome to a perturbation with vitamin D. In monocytes the VDR cistrome comprises more than 10,000 loci, of which a subgroup of a few hundred persistent sites is always occupied (37). These sites seem to be the primary contact points of the human genome with vitamin D and may coordinate the genome's spatio-temporal response to the nuclear hormone.

In THP-1 cells, statistically significant epigenome-wide effects of vitamin D were also described for the binding of the pioneer factors PU.1 (38), CEBPA (30) and GA binding protein transcription factor α (GABPA) (39) as well as for the chromatin organizer CTCF (40). The pioneer factors contribute to the increase in VDR loci after ligand stimulation by helping the receptor accessing its genomic binding sites (Figure 1). Since CTCF majorly contributes to DNA loop formation of the genome into topologically associated domains (TADs) (41), the vitamin D sensitivity of the protein implies that some 500 TADs are triggered by VDR and its ligand. Thus, vitamin D affects the epigenome on multiple levels, such as the binding of VDR and pioneer factors, histone markers, chromatin accessibility, and 3D organization of the nucleus.

## Chromatin Model of Vitamin D Signaling

The chromatin model of vitamin D signaling (24, 42) (Figure 1) was developed on the basis of above described epigenomic data, which had been primarily obtained in THP-1 cells after a stimulation with 1,25(OH)_2_D_3_ for 24 h. The model suggests that a primary vitamin D target gene is modulated in its expression, when the TAD, in which the gene is localized, contains a prominent VDR binding site. This applies to 425 vitamin D sensitive TADs comprising 90% of all target genes in THP-1 cells (40). An additional condition for effective gene regulation is, that the TSS of the target gene as well as the vitamin D-sensitive enhancer have to be located within accessible chromatin (43). DNA looping between the enhancer binding ligand-activated VDR supported by pioneer factors, such as CEBPA and PU.1, and the TSS of a vitamin D target genes changes at both genomic regions H3K27ac and H3K4me3 histone marks as well as chromatin accessibility (Figure 1). Thus, many epigenetic events are required before RNA polymerase II on the TSSs is activated and mRNA synthesis can start.

A meta-analysis of four independent transcriptome-wide datasets of 1,25(OH)_2_D_3_-stimulated undifferentiated THP-1 cells (22, 30, 36, 44) revealed 126 common genes, 72% of which are primary vitamin D targets (45). Nearly all (97%) of these vitamin D target genes are up-regulated and primarily encode for enzymes, receptors and transporters, half of which are located in membranes. Gene ontology analysis indicated the modulation of innate immunity as the most prominent common function of these genes, although this covers <25% of all. Four classes of gene regulatory scenarios, which are based on differential VDR, PU.1, and CEBPA binding to promoter and enhancer regions, can explain the regulation of most (85%) primary vitamin D target genes (45). Interestingly, immune system-related genes are often prominently up-regulated by vitamin D, while genes involved in cellular metabolism are less sensitive to the nuclear hormone. This was confirmed by an independent analysis of vitamin D-triggered TAD classes, where genes that are important for immune function are regulated in a tightly controlled “on/off” modus (37).

## Vitamin D and Immunity

Based on the evolutionary history of nuclear receptors (46, 47), VDR's original function was the regulation of cellular metabolism. This role specialized into the control of calcium homeostasis, when some 400 million years ago species left the ocean and had to improve their calcium-based skeleton, in order to resist to gravitation (48). Although there are no direct effects of vitamin D on bone mineralization, bone-resorbing osteoclasts derive from monocytes, the differentiation of which is controlled by the vitamin D target gene TNF superfamily member 11 (*TNFSF11*, encoding the cytokine RANKL) (49). VDR's tasks in the control of metabolism involves regulating genes mediating energy metabolism, like the glycolytic enzymes fructose-bisphosphatase 1 (*FBP1*) (36) and 6-phosphofructo-2-kinase/fructose-2,6-biphosphatase 4 (*PFKFB4*) (50), as well as in the catabolism of lipophilic intra-cellular molecules, like those encoding for the cytochrome P450 (CYP) enzymes CYP26B1, CYP19A1, and CYP24A1 (51). Both functions supported and enhanced the expansion of the energy demanding immune system as suggested by the concept of immuno-metabolism (52). Moreover, VDR became a critical transcription factor in regulating the expression of genes involved in inflammation and anti-bacterial defense, such as *CD14* (53) and cathelicidin anti-microbial peptide (*CAMP*) (54). Thus, the immune-modulating function of vitamin D is probably evolutionary older than its role in calcium homeostasis.

During hematopoiesis VDR acts together with the pioneer transcription factors PU.1 and CEBPA as a key regulator of myeloid differentiation toward key cells in innate immunity, such as monocytes and granulocytes (55). Furthermore, vitamin D can inhibit the maturation, differentiation and the stimulatory capacity of dendritic cells, which derive from monocytes (56). A profile change of dendritic cells induces the production of regulatory T cells and leads to immunological tolerance. In parallel, vitamin D and its receptor are able to antagonize the pro-inflammatory actions of the transcription factors nuclear factor activated T cells (NF-AT) and nuclear factor κ-light-chain-enhancer of activated B cells (NF-κB) in T cells (57). In this way, vitamin D reduces autoimmunity, such as the onset and progression of multiple sclerosis (58), as well as chronic inflammation, such as in inflammatory bowel disease (59).

Most cells of the immune system have a rapid turnover, which enables them to respond more flexible to environmental changes than other cell types of the human body. For example, monocytes coordinate not only inflammatory pathways, but also control *via* in their differentiated forms, macrophages and dendritic cells, metabolic pathways, and general stress responses. Cellular perturbations, such as an encounter of immune cells with an antigen, affect *via* signal transduction cascades the epigenome. For example, most inflammatory lesions are initiated by monocyte-derived macrophages, the altered gene expression profile of which is based on changes of their epigenome in response to extra-cellular signals (Figure 2C). Moreover, the differentiation process of monocytes to macrophages (or dendritic cells) is based on epigenome changes in response to contacts with antigens. Such a subtype specification is also referred to as trained immunity, as demonstrated by studies of the BLUEPRINT consortium (www.blueprint-epigenome.eu) (60, 61). This rather short-term epigenetic memory monitors the close relationship between immune challenges and effects on chromatin. Epigenetic memory prepares innate immune cells for a possible next microbe encounter (62). In the context of these immunological processes, high affinity receptors for lipophilic signaling molecules, such as VDR and other members of the nuclear receptor superfamily, are in a prime position sensing environmental changes and other signals with a potential of creating cellular stress. Thus, VDR and its ligand are predestined for modulating the process of recording epigenetic memory in innate immunity (63) (Figure 2C).

## *In vivo* Response of Immune Cells to Vitamin D

The chromatin model and the suggested regulatory scenarios of primary vitamin D target genes had been previously developed based on the THP-1 *in vitro* cell system, but are supposed to apply also to other VDR expressing tissue and cell types. This should include *in vivo* situations, such as PBMCs obtained from vitamin D_3_ treated individuals (23). Human supplementation studies allowed the assessment of vitamin D's molecular action under *in vivo* conditions. The studies were carried on PBMCs isolated from participants before and after the long-term (5 months) trial VitDmet (64–67) [NCT01479933, which applied daily vitamin D_3_ supplementation (0–80 μg)] and the short-term (2 days) trial VitDbol (16, 23, 68, 69) [NCT02063334, which used a single vitamin D_3_ bolus (2,000 μg)]. Chromatin and RNA had been immediately isolated from PBMCs, i.e., without any *in vitro* culture, for the assessment of chromatin accessibility [using FAIRE-quantitative polymerase chain reaction (qPCR) and FAIRE-seq] and mRNA expression [using qPCR and RNA sequencing (RNA-seq)]. The changes of molecular parameters, such as the expression of vitamin D target genes or the accessibility of vitamin D-triggered chromatin regions, were related to fold changes in 25(OH)D_3_ serum levels, in order to rank the individuals based on their vitamin D responsiveness (64, 67). The vitamin D response index (15) is proportional to this ranking and segregates the study participants into high, mid, and low responders. Interestingly, the vitamin D response index is a parameter that is independent of the vitamin D status, i.e., of 25(OH)D_3_ serum levels. The vitamin D status is a dynamic parameter and depends on season, diet and supplementation, while the vitamin D index is static, i.e., it is an intrinsic property that is assumed not to change during a person's lifetime. Accordingly, both VitDmet (pre-diabetic elderly participants) and VitDbol (healthy young subjects) agreed on that some 25% of the analyzed cohorts are low responders. These individuals should be supplemented with higher daily vitamin D_3_ doses than high responders. Thus, instead of population-based recommendations for vitamin D_3_ supplementation there should be personalized recommendations in order to reach a vitamin D status that is optimized for an individual's health protection.

PBMCs are a mixture of monocytes, T and B cells, of which monocytes seem to be the most vitamin D-responsive component (6). Based on transcriptome-wide investigations performed with PBMC samples of five participants of the VitDbol study, a vitamin D_3_ bolus significantly changed within 24 h the expression of 702 genes (16, 17). Importantly, 181 (26%) of these genes (such as *CDKN1C, CEBPB, CD14*, and *DENND6B*) were already known in THP-1 cells as vitamin D targets (36); i.e., the *in vivo* response of PBMCs (<10% monocytes) to a vitamin D_3_ bolus resembles to a larger extent the *in vitro* treatment of THP-1 cells with 1,25(OH)_2_D_3_ than expected from the relative cell counts. On the level of the epigenome the overlap between PBMCs and THP-1 cells is even larger, since a vitamin D_3_ bolus significantly affected accessibility of chromatin at 853 genomic loci (23), 87% of which had already been described in THP-1 cells (22). This is exemplified by VDR binding and chromatin opening of the loci of the vitamin D target genes zinc finger MIZ-type containing 1 (*ZMIZ1*, Figure 2A) and myosin IG (*MYO1G*, Figure 2B) under *in vitro* (THP-1) and *in vivo* (PBMC) conditions, respectively. However, the comparison of both cellular systems also indicates that not all genomic regions respond in the same way to vitamin D stimulation. Nevertheless, PBMCs and THP-1 cells show a better overlap on the level of the epigenome than on the transcriptome. Thus, the principles of the chromatin model of vitamin D signaling, which were formulated on the basis of *in vitro* cultured THP-1 cells, may be extrapolated to PBMCs and the *in vivo* situation. Interestingly, data from *in vivo* challenged PBMCs highlighted the human leukocyte antigen (HLA) cluster in chromosome 6 to have a high density of vitamin D-sensitive chromatin regions (23) as well as the genes *HLA-A* and *HLA-C* as vitamin D targets encoding for class I major histocompatibility complex proteins (16). Thus, the HLA cluster serves as a “hotspot” of vitamin D's physiological activity.

## Concluding Hypothesis

Vitamin D is a molecule that is able to modulate *in vitro* as well as *in vivo* the epigenome of immune cells, in particular of monocytes and their differentiated subtypes. In parallel, the rather recently discovered process of trained immunity (70) implies that immune cells memorize challenges, to which they are exposed in their rather short lifespan, in form of changes of their epigenome leading to subtype specification (Figure 2C). By combining these two observations, it is tempting to hypothesize that a large part of the immune-related effects of vitamin D are due to a modulation of the epigenomic programing of monocytes, macrophages, and dendritic cells during their differentiation into subtypes. For example, the HLA cluster, which comprises the highest density of immunologically important genes (71), may be programmed differently in the presence of vitamin D than in its absence. Thus, the efficiency of the epigenetic memory effect of trained immunity should be best at an optimized vitamin D status when vitamin D signaling functions best. Thus, personalized vitamin D_3_ supplementation may support proper epigenetic programming of immune cells throughout hematopoiesis as well as during antigen encounter. In conclusion, the recently discovered epigenome modulation function of vitamin D (24) is essential for understanding the physiological impact of the nuclear hormone.

## Author Contributions

The author confirms being the sole contributor of this work and has approved it for publication.

### Conflict of Interest Statement

The author declares that the research was conducted in the absence of any commercial or financial relationships that could be construed as a potential conflict of interest.

## Abbreviations

1,25(OH)_2_D_3_: 1α,25-dihydroxyvitamin D_3_
25(OH)D_3_: 25-hydroxyvitamin D_3_
BRD7: bromodomain containing 7
CAMP: cathelicidin antimicrobial peptide
CD14: CD14 molecule
CDKN1C: cyclin dependent kinase inhibitor 1C
CEBP: CCAAT/enhancer binding protein
ChIP-seq: chromatin immunoprecipitation sequencing
CTCF: CCCTC-binding factor
CYP: cytochrome P450
DENND6B: DENN domain containing 6B
FAIRE-seq: formaldehyde-assisted isolation of regulatory elements sequencing
FBP1: fructose-bisphosphatase 1
GABPA: GA binding protein transcription factor α
HLA: human leukocyte antigen
KDM6B: lysine demethylase 6B
MYO1G: myosin IG
NF-AT: nuclear factor activated T cells
NF-κB: nuclear factor κ-light-chain-enhancer of activated B cells
PBMCs: peripheral blood mononuclear cells
PFKFB4: 6-phosphofructo-2-kinase/fructose-2,6-biphosphatase 4
PU.1, purine-rich box 1: Spi-1 proto-oncogene (official gene symbol: SPI1)
qPCR: quantitative polymerase chain reaction
RNA-seq: RNA sequencing
TAD: topologically associated domain
TNFSF11: TNF superfamily member 11
TSS: transcription start site
VDR: vitamin D receptor
ZMIZ1: zinc finger MIZ-type containing 1.
